# The historical roots and seminal research on health equity: a referenced publication year spectroscopy (RPYS) analysis

**DOI:** 10.1186/s12939-019-1058-3

**Published:** 2019-10-15

**Authors:** Qiang Yao, Xin Li, Fei Luo, Lianping Yang, Chaojie Liu, Ju Sun

**Affiliations:** 1 School of Political Science and Public Administration, Wuhan University, Wuhan, 430072 Hubei China; 2 School of Psychology and Public Health, La Trobe University, Melbourne, VIC 3086 Australia; 3 School of Information Management, Wuhan University, Wuhan, 430072 Hubei China; 4 Union Hospital, Tongji Medical College, Huazhong University of Science and Technology, Wuhan, 430022 Hubei China; 5 School of Public Heath, Sun Yat-sen University, Guangzhou, 510275 Guangdong China; 6 Institute of Health, Wuhan University, Wuhan, 430071 Hubei China

**Keywords:** Health equity, Historical roots, Milestone works, Reference publication year spectroscopy (RPYS)

## Abstract

**Background:**

Health equity is a multidimensional concept that has been internationally considered as an essential element for health system development. However, our understanding about the root causes of health equity is limited. In this study, we investigated the historical roots and seminal works of research on health equity.

**Methods:**

Health equity-related publications were identified and downloaded from the Web of Science database (*n* = 67,739, up to 31 October 2018). Their cited references (*n* = 2,521,782) were analyzed through Reference Publication Year Spectroscopy (RPYS), which detected the historical roots and important works on health equity and quantified their impact in terms of referencing frequency.

**Results:**

A total of 17 pronounced peaks and 31 seminal works were identified. The first publication on health equity appeared in 1966. But the first cited reference can be traced back to 1801. Most seminal works were conducted by researchers from the US (19, 61.3%), the UK (7, 22.6%) and the Netherlands (3, 9.7%). Research on health equity experienced three important historical stages: origins (1800–1965), formative (1966–1991) and development and expansion (1991–2018). The ideology of health equity was endorsed by the international society through the World Health Organization (1946) declaration based on the foundational works of Chadwick (1842), Engels (1945), Durkheim (1897) and Du Bois (1899). The concept of health equity originated from the disciplines of public health, sociology and political economics and has been a major research area of social epidemiology since the early nineteenth century. Studies on health equity evolved from evidence gathering to the identification of cost-effective policies and governmental interventions.

**Conclusion:**

The development of research on health equity is shaped by multiple disciplines, which has contributed to the emergence of a new stream of social epidemiology and political epidemiology. Past studies must be interpreted in light of their historical contexts. Further studies are needed to explore the causal pathways between the social determinants of health and health inequalities.

## Background

Health equity was a cornerstone of the Millennium Development Goals (MDGs) and a major goal in the Sustainable Development Goals (SDGs) [1]. It has been endorsed by many international and national agencies as an essential element in health system development [2–4]. There is consensus in the international community that health is a fundamental human right [5], and health equity reflects social fairness and justice [6, 7]. Inequity in health can bring detrimental effects on economic development, social vibrancy, and national security [8].

The concept of health equity involves multiple dimensions. In the literature, “health disparity”, “health inequality” and “health inequity” are often used interchangeably [8]. They may measure differences in health system structure (e.g. availability of financial and human resources), health care delivery (e.g. accessibility and quality of care), or health care outcomes (e.g. individual and population health) across populations. Increasingly, researchers agree that health equity is a more appropriate term for defining health system development goals. This is because inherent biomedical differences in health exist among populations; but health is also determined by socioeconomic factors and the differences in health outcomes caused by these modifiable factors are unacceptable. Therefore, the allocation of health care resources and services should be prioritized toward the socioeconomically disadvantaged. These people are likely to suffer from more health problems and need more care. In health care, equity indicates “equal access to available care for equal need, equal utilization for equal need, and equal quality of care for all” [9]. In other words, health inequity measures needs-adjusted inequality in health [10, 11]. Clearly, the concept of health equity is primarily based on the value judgements of morality, justice and human right norms. The most concise and accessible definition of health inequity was articulated by Whitehead in the early 1990s and Braveman in 2006 as “*the systematic, unnecessary, potentially avoidable differences in health or the major socially determined influences on health between groups of people who have different relative positions in social hierarchies according to wealth, power, or prestige, which can be shaped by policies*” [11, 12]. Unfortunately, debates about what differences are unnecessary, avoidable, unfair and unjust will never disappear.

Intellectual history is the foundation for studying an area and is helpful for the researcher to understand the key concepts and comprehensive development process of a research area. It gives researchers an insightful perspective into what their work positions and how their work fits into the overall area. The term “health equality” first appeared in the article “Equality and Health” written by Meltsner in 1966 [13]. This article is commonly accepted as the first published health equity study in several bibliometric studies [14–16], despite some differing personal opinions [17, 18]. However, as a multidisciplinary concept with a long history, the theoretical roots of the term “health equality” can be traced back far before the year of 1966 and has a far-reaching theoretical and practical history. Health equity-related studies had already emerged before the appearance of the terms. At present, the origins or intellectual roots of the concept of health equity is still unclear. A better understanding of the historical context of health equity will render a refreshing perspective on how it all started, in which direction the research field is moving, and what gaps in research need to be filled.

In this study, we investigated the historical roots of health equity studies through identifying the seminal works of relevant research and analyzing publication and referencing trends using the method of Reference Publication Year Spectroscopy (RPYS) proposed by Marx et al. in 2013 [19]. Such a study is necessary to help researchers develop a better understanding of the origin and evolutionary pathway of health equity studies, which is essential for an adequate interpretation of previous studies, the articulation of new research questions and the adaptation of methodological approaches.

## Methods

We performed a RPYS [19] analysis on health equity studies. It quantified the impact of studies in the area of health equity by analyzing the cited references before and after the term(s) appeared [20]. RYPS assumes that a new research area usually evolves on the basis of previous discussions among researchers in the scientific community; and the relationship of current research to past literature plays a significant role, which is usually expressed in the form of citations [21]. The premise of citation theory is that the more frequently cited scientific publications contribute more to the advancement of knowledge [19]. A citation analysis provides insights into the historical context of science. It offers the opportunity for researchers to trace the origin of a new research area even before its concept and terms are formalized.

RPYS has been successfully used to detect the historical roots, milestone works and evolutionary pathways of a number of research areas, ranging from philosophy to medicine, computer science and climate change [19, 21–32]. For example, RPYS analyses revealed that Garfield is one of the pioneers and most influential researchers in bibliometric studies, and his work was influenced by that of Shepard [32]. To the best of our knowledge, no RPYS studies have ever been conducted in the area of health equity research.

### Data source

**Fig. 1:**
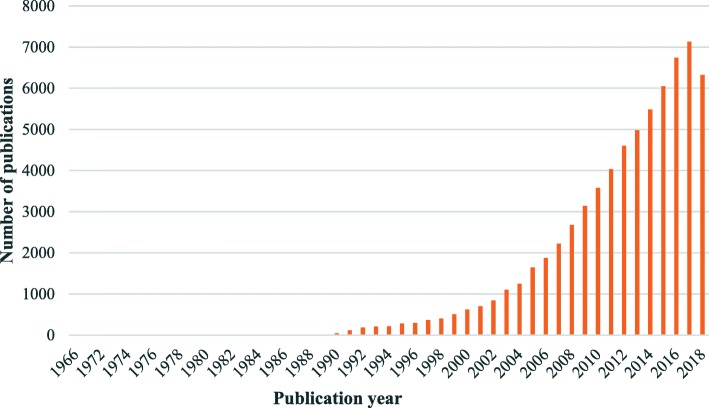
Distribution of publications on health equity from 1966 to 2018

Data for this study, including both publications and their cited references, were extracted from the Web of Science (WoS) core collection using the search strategy “TS=((Health OR healthcare) AND (Equit* OR Equalit* OR Inequit* OR Inequalit* OR Disparit*)) AND PY=1900-2018 AND DOCUMENT TYPES=(Article OR Proceedings Paper OR Review) AND Indexes=SCI-EXPANDED, SSCI, A&HCI”. A total of 67,739 publications from 1966 to 2018 were retrieved on 30th October 2018 (Fig. 1). Full records and cited reference lists (2,521,782 references published from 1800 to 2018) were downloaded for data analyses.

### Data analysis

RPYS was performed to detect the historical roots and important works within the health equity literature. The RPYS analysis assumes that the publication years of the references cited in the literature are not evenly represented; instead, references published in certain years are particularly frequently cited, which appear as distinct peaks in the frequency distribution curves over reference publication years (RPYs). This is usually caused by some important works in the development of the research.

We followed the two-step approach in RPYS [19, 20] using the programs *rpys.exe* and *yearcr.exe* developed by Marx et al. recently [22].

#### Step one - identifying important RPYs

The total number of cited references for each RPY and its deviation from the adjacent five-year (covering the two previous and two following years) median were calculated. The significant peaks across RPYs were identified through deviations of the number of cited references (DoNCR) from the adjacent five-year median using the following rules: (1) For the many small peaks prior to 1950, only those exceeding the upper limit of the 95% confidence interval (CI) of DoNCR for the small peaks were considered significant. Two historical periods were identified, with an upper 95% DoNCR CI limit of 12.18 for 1800–1899 and 35.20 for 1900–1949; (2) All peaks appeared outstanding after 1950 and were identified as significant. This resulted in a total of 21 peaks.

#### Step two - identifying important works within the peaks in RPYs

The importance of a reference was measured by the number of citations it attracted and its percentage to the total citations of all references published in the same RPY as the assessed reference. Previous studies showed that citation patterns evolved over the years, changing from a concentration on some single publications in the 19th and early twentieth century to a focus on the most recent publications since late twentieth century [19, 20]. We followed the same rule in identifying the seminal works: the most cited reference for each peak year prior to 1950 and references with a higher frequency of citation for the years after 1950 compared to those of the cited references published in the preceding and following two years (excluding the years with peaks). Since citations have become increasingly dispersed in recent years, the references with a citation exceeding the mean of the highest cited references published in the preceding and following two years (excluding the years with peaks) were retained for the RPYs since 1950. A total of 36 seminal works were identified.

The research team reviewed the identified seminal works. Those which provided non-specific methodological advice or with relatively low citations and weak relevance to health equity were further excluded. This resulted in a final list of 31 seminal works distributed in 17 peaks (four peaks without seminal works related to health equity) on health equity research. The influence of these seminal works was discussed under the context of the origin and development of health equity research categorized in line with the growth trend and disciplinary distributions of relevant publications.

The division of the development stages for a research area is generally based on the origin of terms and the growth in the speed of publications [27]. In this study, the period before the appearance of health equity terms was regarded as the origins stage. The stable growth of publications using health equity terms (including inequity, inequality and disparity) was labelled the formative stage. This was followed by a rapid development stage that featured exponential growth in the number of publications and an expansion stage that featured a plateau in the number of publications.

## Results

Meltsner published the first article using the term “health and equality” in 1966. The growth of health equity studies was slow initially, with far less than 100 relevant publications each year. Since 1990, studies on health equity started to accelerate. The annual volume of publications on health equity eventually exceeded 1000 in 2003 and continued to grow until reaching the current level of 7132 in 2017 (Fig. 1**).** From 1900 to 2018, the majority were published in public health (e.g. 38.56% on public environmental occupational health) and health services (e.g. 13.56% on health care services and 8.73% on general internal medicine) journals, followed by social science journals (e.g. 6.06% in biomedical social sciences and 2.99% on social sciences other topics).

**Fig. 2:**
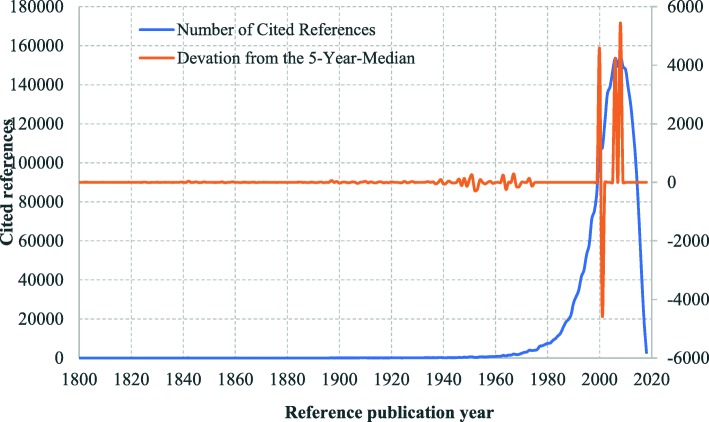
Referenced Publication Years Spectroscopy of Health Equity (1800–2018). Notes:Distribution of the cited references across the reference publication years 1800–2018: Whereas the blue line shows the distribution of the number of cited references across the publication years, and the orange line shows the absolute deviation of the number of cited references in one year from the median for the number of cited references in the two previous, the current and the two following years

**Fig. 3:**
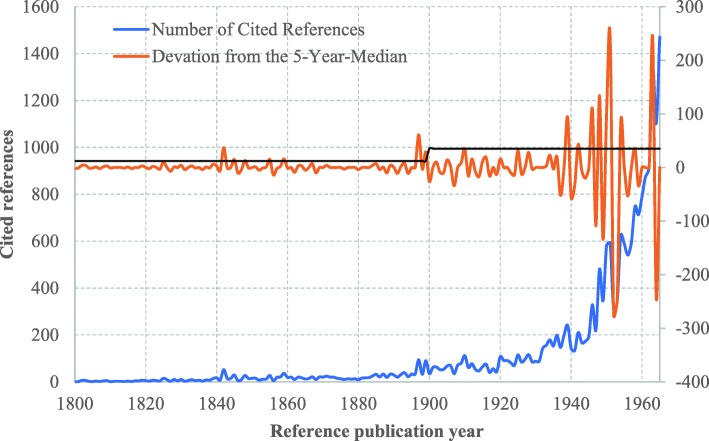
Referenced Publication Years Spectroscopy of Health Equity (1800–1965). Notes:The peaks with seminal works appeared in 1842, 1845, 1897, 1899, 1939, 1946, 1948, 1950, 1951, 1954, 1958 and 1963. The peaks of 1848, 1855, 1859, and 1942 were without seminal works related to health equity

**Fig. 4:**
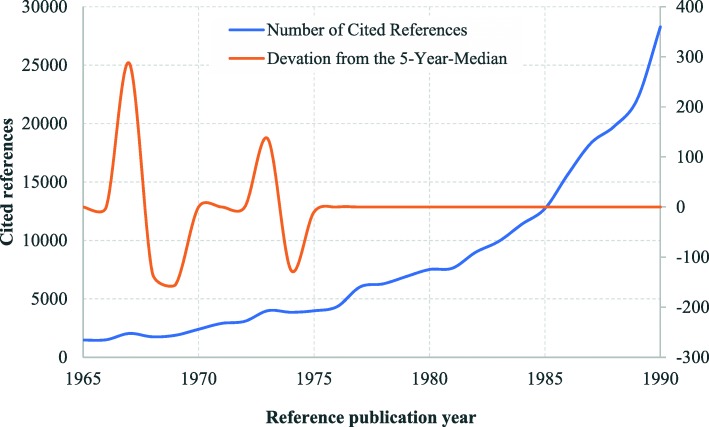
Referenced Publication Years Spectroscopy of Health Equity (1966–1990). Notes:The peaks appeared in 1967 and 1973

**Fig. 5:**
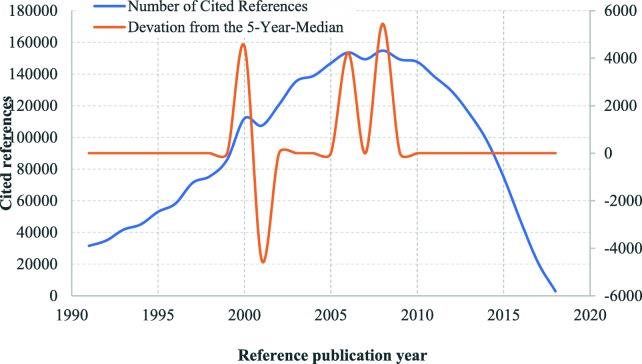
Referenced Publication Years Spectroscopy of Health Equity (1991–2018). Notes:The peaks appeared in 2000, 2006 and 2008

The cited references of health equity studies can be traced back to 1801. The distribution of cited references by RPYs indicated a three-stage development in health equity studies **(**Fig. 2**)**: origins (pre-1965), formative (1966–1990), and development and expansion (1991–2018). There were many small peaks in DoNCR in the origins stage, compared with a few major peaks occurring in the formative and development and expansion stages. In total, we identified 17 significant peaks with seminal works: 12 in the origins stage **(**Fig. 3**)**, 2 in the formative stage **(**Fig. 4**)**, and 3 in the development and expansion stage **(**Fig. 5**)**.

Approximately 31 seminal works contributed to the 17 peaks (Table 1). Most of the seminal works were conducted by researchers in the US (19, 61.3%), the UK (7, 22.6%) and the Netherlands (3, 9.7%). The first seminal work conducted by Chadwick from Great Britain appeared in 1942, which revealed variations in life expectancy associated with social class. It signals the emergence of health equity studies. The ideology of health equity was eventually endorsed by the international society through the World Health Organization (WHO, 1946) declaration based on the foundational works of Chadwick (1842), Engels (1945), Durkheim (1897) and Du Bois (1899). Since then, the scope of health equity studies has been extended from a focus on mortality and morbidity to a view on health and wellbeing, from a wealth-related perspective to perspectives of a variety of social classifications (e.g. social stigma and discriminations), from quantitative descriptions to qualitative understanding of root causes, and from a national approach to an international approach. The WHO Commission on Social Determinants of Health (2005) provided a systematic framework for health equity studies.

**Table 1:** Summary of the most frequently cited references (1800–2018) in health equity studies

No	Reference Publication Year	Authors, Title and Source	Country	Citation of References			Brief summary
				Number (%) in the Peak Year		Total in Google Scholar^a^	
1	1842	Chadwick E. Report on the Sanitary Condition of the Labouring Population of Great Britain: supplementary report on the results of special inquiry into the practice of interment in towns (Vol. 1). HM Stationery Office.	Great Britain	40	(76.92)	885	The report highlighted variations in life expectancy associated with class or residency in statistics: middle-class people lived longer and healthier because they could afford to pay for sewage removal and fresh water connection to homes. The argument about the importance of sanitary conditions contributed to the passing of the Public Health Act and the Public Health Bill.
2	1845	Engels F. The condition of the working class in England. Leipzig: Otto Wigand.	Great Britain	16	(55.18)	4560	This book proposed that the industrial revolution made workers worse off: industrial workers had lower income, worse living environments and poorer health than their pre-industrial peers.
3	1897	Durkheim, É. (1897). Le suicide.	France	58	(61.69)	1724	This book studied suicide and its social causes: suicide was found to be associated with nationality, religion, age, sex, race, marital status, economic status, educational level, family size, place of residence, and exposure to war conflicts.
4	1899	Du Bois WEB & Eaton I. The Philadelphia Negro: a social study (No. 14). Published for the University.	USA	28	(31.10)	2545	This book presented the first statistical social study on a black community in the US: “Negro problem” was ostensibly “not one problem, but rather a plexus of social problems” caused by whites’ enforcement of racial discrimination and a provision of unequal opportunity.
5	1939	Faris REL & Dunham HW. Mental disorders in urban areas: an ecological study of schizophrenia and other psychoses. Oxford, England: Univ. Chicago Press	USA	50	(20.73)	2327	This book revealed a close relationship between mental disorders and the ecological structure of a city using ecological mapping: the distribution of schizophrenia was associated with sex, race, income, social relationship and home location.
6	1946	World Health Organization (WHO). Constitution of the World Health Organization.	World Health Organization	56	(16.99)	2151	The preamble of the WHO constitution defines health as “a state of complete physical, mental and social well-being and not merely the absence of disease or infirmity” and proposes that “the enjoyment of the highest attainable standard of health is one of the fundamental rights of every human being without distinction of race, religion, political belief, economic or social condition”.
7	1948	United Nations (UN) General Assembly. Universal declaration of human rights. UN General Assembly.	United Nations	83	(17.34)	1078	The declaration sets health as a fundamental human right and proposes that “everyone has the right to a standard of living adequate for the health and well-being of himself and of his family”, and “motherhood and childhood are entitled to special care and assistance”.
8	1950	Robinson WS. Ecological correlations and the behavior of individuals. American Sociological Review, 15.	USA	70	(12.05)	5720	This study established ecological correlations (also spatial correlations) of behaviors of individuals: ecological correlations can measure the strength of a relationship and correlations at the group level can be much higher than those at the individual level.
9	1951	Cronbach LJ. Coefficient alpha and the internal structure of tests. Psychometrika, 16(3), 297–334.	USA	77	(13.04)	38,409	This paper suggested the use of Cronbach’s alpha (or coefficient alpha) for measuring reliability (internal consistency) of psychometric instruments/scales, which has since been widely adopted in studies in psychology, social sciences, business, nursing, and other disciplines.
10	1951	Talcott P. The social system. Routledge.	USA	58	(9.81)	24,703	This book presents a classic study on social systems and the “Theory of Action”, which laid a robust foundation for social systems theories and provided a theoretical framework for studies in a variety of areas, including medical practice, kinships and role-socialization, psychological relationships, and religious organization.
11	1954	Festinger L. A theory of social comparison processes. Human relations, 7(2), 117–140.	USA	71	(11.36)	19,949	This book extends the previous proposed theory of social comparison to studies appraising and evaluating abilities and opinions: how individuals evaluate their own opinions and abilities by comparing themselves to others in order to reduce uncertainty and learn how to define self.
12	1954	Allport GW, Clark K & Pettigrew T. The nature of prejudice.	USA	61	(9.76)	30,882	This book redefines intergroup relationships and prejudice: the Allport’s Scale was developed to measure prejudice ranging from ant locution to genocidal extermination.
13	1958	Kaplan EL & Meier P. Nonparametric estimation from incomplete observations. Journal of the American statistical association, 53(282), 457–481.	USA	64	(8.57)	55,002	This study developed the Kaplan–Meier estimator, a nonparametric estimation method, for analyzing the survival function using incomplete lifetime data. It has fewer assumptions and is simpler compared with the parametric methods, which is particularly useful for data with a ranking but no clear numerical interpretations.
14	1958	Hollingshead AB & Redlich FC. Social class and mental illness: Community study.	USA	57	(7.62)	8933	This research monograph found a true link between social class and the distribution of mental illness being treated and the place of patients being treated in populations.
15	1963	Goffman *E. stigma*. Notes on the Management of Spoiled Identity. New York: Simon and Shuster.	USA	215	(16.00)	34,212	This book discusses how social stigma (e.g. social deviation, physical or mental defects) is developed and denies full social acceptance of some groups and individuals.
16	1963	Katz S. Studies of illness in the aged. The index of ADL: a standardized measure of biologic and psychologic function. JAMA, 185, 94–99.	USA	117	(8.71)	10,761	This study developed the ADL index measuring primary biological and psychosocial functions, and proposed the use of the ADL index as a tool for assessing the outcomes of clinical interventions which can guide clinical practices and help improve our understanding about aging.
17	1963	Arrow KJ. Uncertainty and the Welfare Economics of Medical Care. The American Economic Review, 53(5):941–73.	USA	110	(8.19)	8877	This paper summarized the special characteristics of the medical care market: differences between the medical care market and a typical competitive market. It highlighted the uncertainty and welfare economics of medical care.
18	1967	Glaser BG, Strauss AL & Strutzel E. The discovery of grounded theory; strategies for qualitative research. Nursing research, 17(4), 364.	USA	390	(19.12)	1516	This book describes the grounded theory, a new interpretive approach (compared with the positivism approach) to qualitative studies on emerging social and cultural issues. According to the grounded theory, new theories arise from qualitative data. This approach has since been widely endorsed by the international research community.
19	1967	Antonovsky A. Social class, life expectancy and overall mortality. The Milbank Memorial Fund Quarterly, 45(2), 31–73.	Israel	115	(5.64)	1019	This study revealed class influences on one’s chance of alive: the largest class difference existed in the middle years of life.
20	1973	Kitagawa EM & Hauser PM. Differential mortality in the United States: A study in socioeconomic epidemiology.	USA	264	(6.62)	2095	This book presents findings of a study that revealed differential mortality across a broad spectrum of social and economic factors (including age, sex, education, income, occupation, race, marital status, parity, nativity, and geographic classifications): lower socioeconomic status is associated with higher risks of dying and lower life expectancy.
21	1973	Andersen R & Newman JF. Societal and individual determinants of medical care utilization in the United States. The Milbank Memorial Fund Quarterly. Health and Society, 95–124.	USA	229	(5.75)	3574	This study provided a widely-accepted framework for operationalizing assessment of “equitable distribution of health services”: health care utilization is determined by both need factors and enabling factors at the individual, household and societal levels.
22	2000	Berkman LF & Kawachi I. Social Epidemiology.	USA	1487	(1.33)	2856	This is the first book of social epidemiology, describing a new sub-discipline in the field of epidemiology that focuses particularly on the effects of social class on health.
23	2000	Office of Disease Prevention and Health Promotion, US Department of Health and Human Services: Healthy People 2010. http://www.health/gov/healthypeople/.	USA	1303	(1.16)	1728	This US governmental document depicts two overarching goals: to enhance life expectancy and the quality of life and to eliminate health disparities between different segments of the population. It contributed to the development of various models measuring disparities.
24	2003	Smedley BD, Stith AY & Nelson AR. Unequal treatment: Confronting racial and ethnic disparities in health care.	USA	2289	(1.69)	7003	This Institute of Medicine report warned that racial and ethnic minorities received lower quality healthcare than whites, and the bias, prejudice and stereotyping of healthcare providers might have contributed to the unequal treatment. A comprehensive multi-level strategy was proposed to eliminate these disparities.
25	2005	Marmot M. Social determinants of health inequalities. The lancet, 365(9464), 1099–1104.	UKWHO	582	(0.40)	8790	This paper describes the tasks of the WHO Commission on Social Determinants of Health, addressing social factors leading to ill health and health inequity. It not only reviewed the existing body of knowledge but also raised societal debates on health inequalities within and between countries.
26	2006	Galobardes B, Shaw M, Lawlor DA, Lynch JW & Smith GD. Indicators of socioeconomic position (part 1). Journal of Epidemiology & Community Health, 60(1), 7–12.	UKUSA	605	(0.39)	1457	This study presents a comprehensive list of indicators measuring socioeconomic position (SEP) that were commonly used in health research (education, income, housing characteristics, occupation, occupational based measures, proxy indicators, composite indicators, and area level measures): the theoretical basis, measurement, interpretation, strengths and limitations of each indicator.
27	2006	Wilkinson RG & Pickett KE. Income inequality and population health: a review and explanation of the evidence. Social science & medicine, 62(7), 1768–1784.	UK	487	(0.32)	1536	This paper reviewed the relationship between income inequality and inequality in population health, and proposed that income distribution is a convenient and widely applicable measure for socioeconomic stratifications.
28	2006	Van Doorslaer E, Masseria C, & Koolman X. Inequalities in access to medical care by income in developed countries. Canadian medical association journal, 174(2), 177–183.	Netherlands,UK, OECD countries	341	(0.22)	825	This study examined inequity in the use of physician services (in 2000) in the 21 OECD countries using data extracted from the national household surveys. The study found that primary care (general practice) was pro-poor or distributed fairly equally, while specialist care tended to be pro-rich.
29	2008	Marmot M, Friel S, Bell R, Houweling TA, Taylor S & Commission on Social Determinants of Health. Closing the gap in a generation: health equity through action on the social determinants of health. The Lancet, 372(9650), 1661–1669.	UK, WHO	1929	(1.25)	4063	This paper summarized findings of the WHO Commission on Social Determinants of Health, and called for all governments to lead actions addressing social determinants of health with an aim to achieve health equity. The paper also set key action areas associated with daily living conditions and their underlying structural drivers.
30	2008	Mackenbach JP, Stirbu I, Roskam AJR, Schaap MM, Menvielle G, Leinsalu M & Kunst AE. Socioeconomic inequalities in health in 22 European countries. New England Journal of Medicine, 358(23), 2468–2481.	Netherlands, Sweden, Estonia, European Union countries	903	(0.58)	2383	This study compared the magnitude of inequalities in mortality and self-assessed health among the 22 European countries using a regression-based inequality index: inequalities in health varied across the European countries, which were associated with the socioeconomic status of each country.
31	2008	O’donnell O, Van Doorslaer E, Wagstaff W & Lindelow M. Analyzing health equity using household survey data.	Netherlands,World Bank	531	(0.34)	1656	This book provides researchers with a step-by-step practical guide to the measurement of various aspects of health equity: in access to health services, in financial contributions to health systems, and in health outcomes. This led to a more comprehensive approach to monitoring of the trends in health equity, understanding of the causes of health inequities, extensive evaluation of the effects of development programs on health equity, and development of effective policies and programs to reduce inequities in the health sector.

^a^Data from https://scholar.google.com/ (accessed 12 February 2019)

### Origins stage (pre-1965)

In a span of over one and a half centuries (1801–1965), 16 small peaks appeared (in 1842, 1845, 1848, 1855, 1859, 1897, 1899, 1939, 1942, 1946, 1948, 1950, 1951, 1954, 1958 and 1963), exceeding the upper limit of the 95% CI of DoNCR (Fig. 3**).** But no seminal works were identified for the small peaks in 1848, 1855, 1859, and 1942.

#### Origins (1800–1899)

Four significant peak RPYs were identified over the period from 1800 to 1899, each featuring one publication. The four publications were all presented as books/reports, attempting to stratify health conditions according to social classes and groups. Chadwick (1842) from Great Britain [33] used evidence of variations in life expectancy to argue the importance of sanitary conditions and eventually contributed to the passing of the Public Health Act. Engels (1845), also from Great Britain [34], reported the shortage of the benefits of industrialization to the working class and their worse than pre-industrial living conditions. Durkheim (1897) from France [35] discussed suicide associated with residence and other sociodemographic factors. Du Bois & Eaton (1899) from the US [36] were the first to study social and health problems in the black community in the context of racial segregation (details in Table 1). These works examined population health, in particular, environmental and occupational health issues, but did not focus much on healthcare systems, a major concern of today’s health equity studies. However, they unveiled the fact that there was a disproportional distribution of public health problems during the process of social and technological advancement.

#### Early theoretical development (1900–1965)

The period of 1900–1965 laid a critical theoretical and methodological foundation for the latter development of health equity studies. A total of 8 significant peak RPYs were identified for this period, involving 13 seminal works. These works contributed to the development of the “health as a human right” framework, social class theories, methodological approaches to statistical analyses on spatial distribution and social distribution of health, and measurements of health (No.5 to No.17 in Table 1).

The WHO (1946) [37] and the United Nations General Assembly (1948) [38] proposed that, as a fundamental human right, every human being is entitled to the highest attainable standard of health without the distinction of race, religion, political belief, economic or social condition. These works set up the basic tone and essential principles for health equity.

A series of studies conducted by sociologists in the US proposed social class theories, offering insights into how a social system can be described in terms of interactions between actors (Talcott, 1951) [39], how individuals compare with others and attach themselves to a social group (Festinger, 1954), how social groups contact and form prejudice against others (Allport et al., 1954) [40], and how social stigma which denies full social acceptance of some groups and individuals develops (Goffman, 1963) [41]. These theories were widely used in classifying social groups and help explain the underlying reasons for health inequity between socially advantaged and disadvantaged groups.

The unequal distribution of health was studied using a range of approaches. The seminal works included the ecological mapping of schizophrenia (Faris & Dunham, 1939) [42] and exploration of social class differences in the treatment of mental illness (Hollingshead et al., 1958) [6]. Robinson (1950) [43] highlighted the importance of research into the correlations between mean outcomes and mean characteristics of social groups: correlations at the group level were much higher than those at the individual level.

The measurement of health was enhanced by incorporating the perspective of consumers. Cronbach’s alpha (1951) was proposed for testing the reliability (or internal consistency) of instruments measuring patient-reported health outcomes [44]. The Kaplan–Meier estimator (1958) [45] was proposed as a nonparametric estimation method, solving the problem of the statistical testing of data that have a ranking but no clear numerical interpretation. The advancement of statistical methods and psychometric tests boosted the confidence of researchers in using subjective (patient-reported) health indicators. The Katz (1963) instrument measuring independence in activities of daily living (ADL) [46] gained wide acceptance. In addition, people’s understanding about the function of health care systems, in particular in relation to health equity, was significantly improved by the work done by Nobel laureate, Kenneth Arrow, which summarized the special characteristics of the health care market in comparison with a typical competitive market [47].

### Formative stage (1966–1990)

Between 1966 and 1990, two large peak RPYs were identified (Fig. 4). For the first time, the term “health and equality” was used in the literature [13].

Over this period of time, large epidemiological studies were conducted to quantify the health gaps in populations. Two epidemiological studies on social class differences in mortality were identified as seminal works. Antonovsky (1967) [48] revealed that social class influenced one’s chance of being alive and class difference was the largest in the middle years of life. Kitagawa & Hauser (1973) [49], in their study labelled “socioeconomic epidemiology”, confirmed that mortality differentials existed across a broad spectrum of social and economic factors (No.19 to No.20 in Table 1).

Inequity in healthcare services started to attract attention. Both healthcare and other social service systems were examined for their roles in addressing disparities in accessing health care. The Andersen health utilization model published in 1973 [50] provided a widely-accepted framework for operationalizing the assessment of the “equitable distribution of health services”. It proposed that health care utilization is determined by both need factors and enabling factors at the individual, household and societal levels (No.21 in Table 1).

Over this period of time, qualitative research methods also started to gain mainstream recognition in the research community, thanks to the groundbreaking “grounded theory” (Glaser et al., 1967) [51] which provided a systematic methodological approach for researchers to study emerging social and cultural phenomena, including health inequity issues and develop new theories arising from qualitative data (No.18 in Table 1).

### Development and expansion stage (1991–2018)

Three large peaks in RPYs appeared over the years between 1991 and 2018 (Fig. 5). The peak RPYs occurred only when several seminal works made significant co-contributions. A total of 10 seminal works over this period were identified, including two published in the non-peak RPYs (2003 and 2005). Seminal works in this stage featured the formulation of the new discipline “social epidemiology” and system perspectives on healthcare and health equity at national and international levels. Researchers from the UK played a leading role in the seminal works: 50.0% from the UK (5), followed by 40.0% from the US (4) and 30.0% from the Netherlands (3).

#### Rapid development (1991–2005)

Research on health equity entered a period of rapid development between 1991 and 2005. The peak RPY occurred in 2000, accounting for 9.89% of all cited references (1,132,343) published over this period. But not a single publication contributed over 1.33% of the citations, suggesting co-contributions of a number of seminal works. Four seminal works were identified for this period: two fell into the peak RPY (in 2000); the other two were published in 2003 and 2005, respectively (Table 1).

This period witnessed the official endorsement of the “concept and principles of equity in health” by the WHO in the early 1990s and the establishment of “social epidemiology” (Berkman and Kawachi, 2000) [52], a new sub-discipline of epidemiology focusing particularly on the effects of social class on health. The most influential works on health equity conducted at the national system level included “Healthy People 2010” promulgated by the US Department of Health and Human Services (in 2000) [3], and the Institute of Medicine (2003) report [53] on income and race-related disparities in health and healthcare. The US led in the development of national strategies for improving and monitoring the progress of health equity. At the global system level, Marmot’s article “Social Determinants of Health Inequalities” (2005) on behalf of the WHO Commission on Social Determinants of Health [54] emphasized the importance of looking at the problem of health inequity beyond the traditional scope of health care services (No.22 to No.25 in Table 1).

#### Plateaued expansion (2006–2018)

Publications on health equity continued to grow from 2006. But citations of these publications appeared to experience a plateau (the decline of citations since the RPY of 2010 may possibly be offset by citations in future publications). Two peak RPYs were identified. The first peak occurred in 2006, contributing to 12.9% of the citations over this period, compared with 13.0% contributed by the second peak in 2008. Not a single publication contributed over 0.39% of the citations in the first peak RPY and 1.25% in the second peak RPY, respectively. Therefore, several papers in each peak RPY were identified as seminal works (Table 1).

Methods for health equity studies were further refined. Galobardes et al. (2006) [55, 56] presented a comprehensive list of indicators measuring the socioeconomic status of people, including the theoretical basis, measurements, interpretation, strengths and limitations of each indicator. O’Donnell, Van Doorslaer, Wagstaff & Lindelow (2008) [57] developed a technical guideline entitled “Analyzing health equity using household survey data” on behalf of the World Bank, which covers the equity of health systems in structure (e.g. financial contributions), process (e.g. accessibility), and outcomes (income-related health outcomes).

Over this period, two seminal works compared health equity across countries: one among the Organization for Economic Co-operation and Development (OECD) countries (Van Doorslaer et al., 2006) [58]; and the other among the European Union countries (Mackenbach et al., 2008) [59].

This period also featured calls for action on the social determinants of health. Wilkinson and Pickett (2006) [60] proved that greater income inequalities are associated with poorer overall population health. The WHO Commission on Social Determinants of Health report “Closing the gap in a generation: health equity through action on the social determinants of health” (Marmot, 2008) [2] attracted the highest citation rate (1929, 1.25%) over this period (No.26 to No.31 in Table 1).

## Discussions

This study detected the historical roots and seminal works of health equity using RPYS. A total of 17 pronounced peaks in RPYs were identified over the years from 1800 to 2018. These citation peaks are indicative of milestone works in research on health equity: 31 seminal studies. The historical evolutionary path in health equity studies is characterized by three distinctive stages: origins (1800–1965), formative (1966–1991), development and expansion (1991–2018).

The origins stage (1800–1965) is in line with the development of modern public health. Poor sanitation and living conditions of the poor and working class were proved to be associated with high mortality and poor population health, triggering governmental interventions through public health acts. These populations were deemed highly susceptible to infectious diseases in epidemiology, and if not managed properly, could impose serious health risks to the entire society. At this stage, there was not much discussion about unequal access to health care services. Instead, environmental and occupational health and safety for the entire society was the major focus. However, the “Constitution of the World Health Organization” (1946) and the preamble to the United Nations “Universal Declaration of Human Rights” (1948) signals a development in conceptualizing health equity [18]. Since the 1950s, research instruments for studying health equity have been developed, providing theoretical and methodological foundations for research into health equity.

In the formative stage (1966–1990), the terms “health disparity”, “health equality” and “health equity” were formally defined. Sociological studies made a significant contribution to the development of health equity research. This was driven, at least partly, by the grounded theory proposed by Glaser et al. (1967). This stage also features the wide acceptance of social class theories and their use in epidemiological studies. However, the lack of a specified sub-discipline and relevant research methods restricted the growth of health equity studies.

In the third stage (1991–2018), social epidemiology was established with a specific mission to study health equity issues. The concept of health equity was further clarified in the early 1990s [15, 16]. Researchers started to examine health equity issues from national and international perspectives based on the theory of health systems. Driven by the WHO and the World Bank, a consensus on the measurements of the social determinants of health and the methods for decomposing the contributions of socioeconomic factors on health equity was reached. This contributed to a rapid growth in health equity studies. The WHO proposed a global goal for achieving health equity through universal health coverage.

Overall, health equity studies have made a significant contribution in driving the global agenda of “health for all and health in all” [61, 62]. A systems approach beyond the health sector is recognized as essential for achieving health equity [63]. Researchers from the UK, the US, and the Netherlands led the development and growth of research into health equity. This finding is consistent with previous studies [15, 64]. The WHO was the leading agency driving the agenda of health system development with a strong focus on health equity. The efforts of the WHO were supported by its member countries, the World Bank, regional organizations such as the OECD and the European Union, and many non-governmental organizations (NGOs). However, scant studies have been conducted from a political perspective [65, 66], although actions on the social determinants of health are often intensely political [67]. Some researchers have called for increasing attention on socio-political analyses of health equity studies, which will not only offer novel theoretical insights but also methodological innovations [68, 69].

Significant progress has been achieved in measuring health equity [55, 56, 66, 70–75] and decomposing contributions of various determinants of health inequity [76–82]. But there is a lack of understanding of the pathways and mechanisms of how the social determinants of health lead to health inequalities. The existing studies are criticized for a bias towards socioeconomic factors (e.g. income level, social class, educational achievement, and occupational status), midstream factors (e.g. biology, health-related behaviors, environment, and health benefits) and downstream factors (e.g. healthcare services access and use factors) [67]. This has limited the usefulness of health equity studies in political and policy decision making [83–85]. Recently, several theoretical frameworks have been proposed to facilitate research into the causal pathways between upstream social determinants of health (e.g. material, social, political, and cultural circumstances) and health inequity [86–88], such as social disadvantage and life course approaches [89, 90]. The focus of health equity studies has been extended to concerns of a much broader range of socioeconomic and upstream factors, such as poor health services and health outcomes experienced by the sexual minorities [91–93] and those with disabilities and mental health problems [66, 94] as a results of victimization and discrimination compounded by stigma [95, 96]. However, there is a lack of understanding about how these factors interact with other socioeconomic factors [96]. Dover and Belon developed a framework integrating the existing social determinants of health framework and the health services utilization framework [97]. It is expected that more studies will emerge addressing health equity concerns from an economic perspective [98–100]. Despite the progress, health equity studies remain a challenge due to the long and complex causal pathways linking individual and social factors to health and our limited ability to study this topic using randomized control trials [89].

## Conclusions

The historical origins of health equity research can be traced back to the early 1800s and is embedded in the development of public health. It has become a major focus of the sub-discipline of social epidemiology, involving a set of measurements and methodological approaches. Sociological studies made a significant contribution to the development of social epidemiology. The concept and principles of heath equity have now been widely accepted in the international community thanks to the efforts of the WHO. Health equity studies are moving from evidence gathering to the identification of cost-effective policies and governmental interventions. This may lead to the emergence of another sub-discipline “Political Epidemiology” as suggested by Pega and Kawachi from Harvard University [101]. New frameworks and analytic approaches are needed to investigate the causal pathways between the social determinants of health and health inequalities [88, 97].

## Limitations

There are several limitations in this study. Firstly, this RPYS study relied on the WoS collection, similar to previous RPYS studies. The WoS database covers high quality journals. But it is biased toward publications in the English language. However, the RPYS method has a focus on references, which expands the coverage of publications and may also reduce the language bias. Secondly, the RPYS does not analyze citation relationships and networks. Future studies should consider the use of co-citation analyses, bibliographic coupling, and direct citations in consideration of research fronts [102].

## Data Availability

The datasets analyzed during the current study are available in the Web of Science Core Collection, [www.webofknowledge.com].

## Abbreviations

ADL: Activities of daily living
CI: Confidence interval
DoNCR: Deviations of the number of cited references
MDGs: Millennium Development Goals
NGOs: Non-governmental organizations
OECD: Organization for Economic Co-operation and Development
RPY: Reference publication years
RPYS: Referenced publication year spectroscopy
SDGs: Sustainable Development Goals
WHO: World Health Organization
WoS: Web of Science
